# Complete Denture Fabrication Using Digitally Fabricated Copy Dentures for a Patient with Moderate Dementia

**DOI:** 10.1155/2021/9385095

**Published:** 2021-07-24

**Authors:** Korehide Arai, Yuto Tanaka, Shinsuke Matsuda, Tomohiko Okamura, Kazufumi Iwayama, Yoshiaki Ono

**Affiliations:** ^1^Department of Special Care Dentistry, Osaka Dental University Hospital, Osaka, Japan; ^2^Matsuda Oral Appliance, Osaka, Japan

## Abstract

A 91-year-old woman was referred to our hospital with a chief complaint of unsatisfactory fit and pain associated with her complete dentures. She had moderate dementia with difficulty in communication (Mini-Mental State Examination, 16; Barthel Index, 15). The closed impressions and jaw record were taken with the digitally fabricated copy dentures as follows. First, the tissue conditioner was used to correct the poor fit of the old dentures, following which minor occlusal alterations were made. Second, the copy dentures that copied the morphology of the corrected old dentures using three-dimensional (3D) scanner were fabricated with a 3D printer. The new dentures were then fabricated using conventional methods as follows. The impressions were cast and articulated, and the dentures were subsequently processed. This case report documented the following results. First, the acceptance of new dentures appeared to be easier since the new dentures copied the morphology of the familiar dentures digitally. Moreover, the 3D data of the dentures could be used for immediate denture fabrication in case of fracture or loss of the dentures. Second, only two visits were required for taking an impression and delivering the complete dentures. In addition, her old dentures were brought to our dental office by the patient's family after the patient's dinner; immediately after copying the dentures' morphology, the dentures were returned to the patient's family, thus avoiding any disturbance to the patient's eating routine. These reduced the burden on the patient and her family.

## 1. Introduction

Some elderly patients requiring complete denture fabrication are medically frail or are homebound with major limitations in mobility [1]. While some individuals can independently access the dental office, others are institutionalized or require long-term care at home; these individuals might require a dentist to provide care at their place of residence. These facts emphasize the need to reduce the number of visits to dental clinics for fabrication of complete dentures [2]. On the other hand, it is difficult to ensure the safety of dental treatment for these patients. In particular, treatment requiring low-viscosity materials place a heavy burden on the patients with impaired oropharyngeal function [3]. Therefore, in such nondexterous patients, denture modification is preferred over a new denture fabrication. However, temporary denture modifications, such as tissue conditioning with acrylic-based tissue conditioner, lead to several problems, including bond failures to the denture base, accumulation of oral microorganisms, poor tear strength, and hardness changes.

In contrast, digital dental technologies, with the aim of highly safe dental treatment and efficiency, have significantly improved in the last decade [4]. For example, based on the results of an integrative review, no significant difference was found regarding the marginal discrepancy of single-unit ceramic restorations fabricated following digital and conventional impressions [5]. Therefore, in this case report, new dentures were manufactured by incorporating digital dental technology with conventional methods to reduce the burden on edentulous patients with severe dementia and to improve overall safety.

## 2. Case Presentation

The patient was 91 years of age and a female. As the chief complaint, the patient expressed unsatisfactory fit and pain associated with use of the complete dentures. The patient's family desired new dentures for her, as her dentures appeared to fit loosely, and she was unable to eat properly.

For the degree of care, the patient exhibited difficulty in communicating (Mini-Mental State Examination, 16; Barthel Index, 15). Her medical history is as follows: The patient developed symptoms of Alzheimer's-type dementia five years prior, following which she has been under the care of her family. In addition to taking an acetylcholinesterase inhibitor to treat the dementia, the patient had been prescribed antihypertensive and antihyperuricemic drugs.

The patient has a height of 150.0 cm and weight of 47.0 kg.

**Figure 1:**
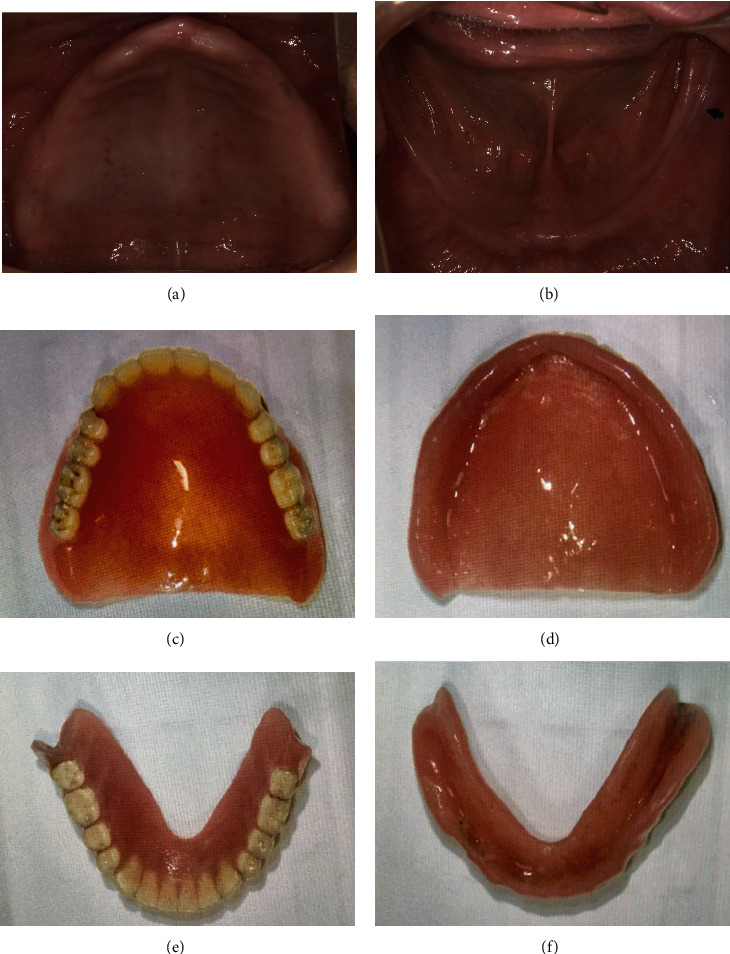
Intraoral findings ((a) upper jaw, (b) lower jaw) and a familiar denture used by the patient ((c) occlusal surface of upper denture, (d) mucosal surface of upper denture, (e) occlusal surface of lower denture, and (f) mucosal surface of lower denture). The arrowhead indicates an ulcer in the left alveolar ridge of the lower jaw.

A home-visit dentist manufactured the complete dentures for the upper and lower jaws two years prior. The following year, she complained of pain in the left alveolar ridge of the mandible and requested a home visit for dental treatment; however, her symptoms and food intake did not improve. She was referred to our hospital for the fabrication of new dentures.  Figure 1 shows the oral findings (Figures 1(a) and 1(b)) and the old dentures (Figures 1(c)–1(f)). An ulcer was noted in the left alveolar ridge of the mandible, with severe bilateral ridge resorption (Figure 1(b)).

## 3. Procedure

**Figure 2:**
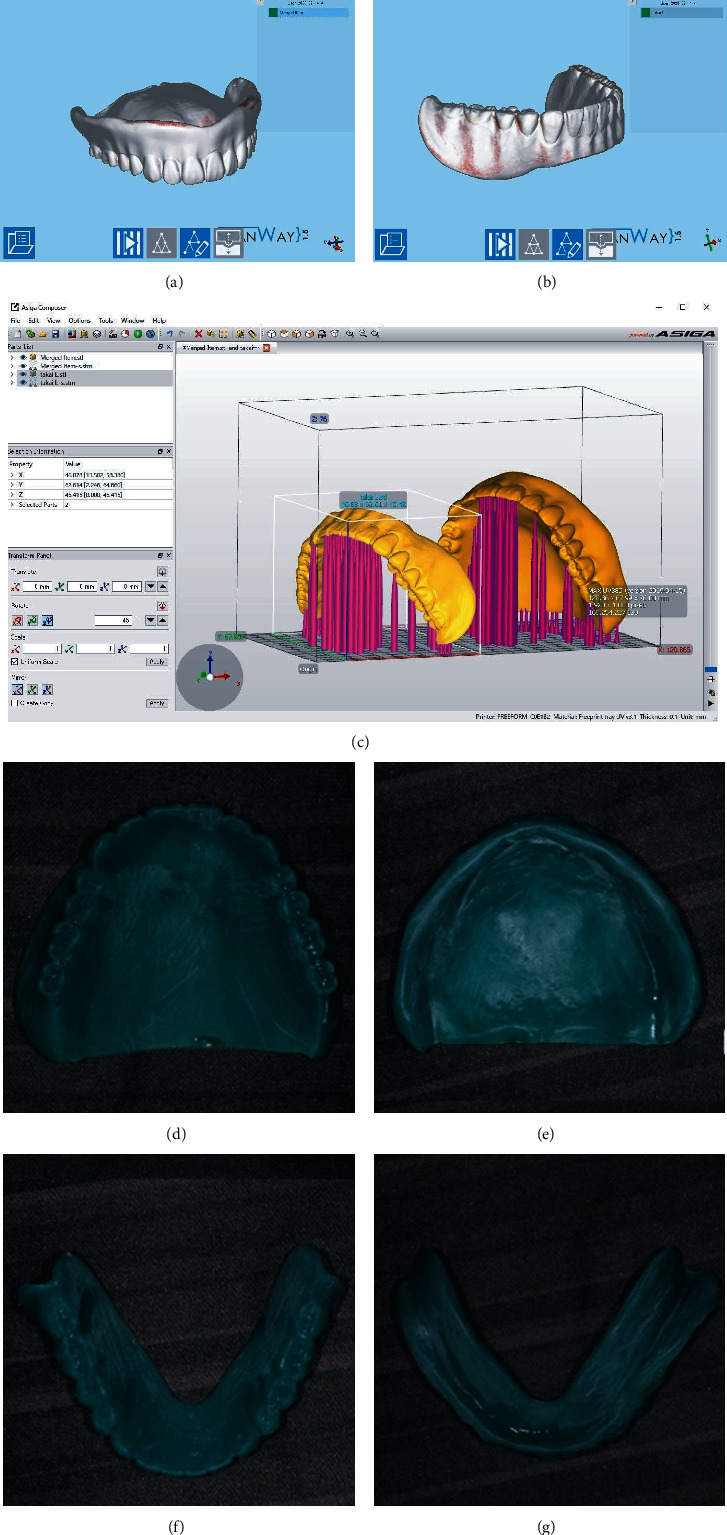
Optical impressions recorded for the upper (a) and lower (b) dentures with an optical scanner. The scanned data were converted into standard tessellation language (STL) data and exported to a three-dimensional (3D) printer interface software, after which the support bars were set (c). A 3D printer using dental photocurable resin fabricated the copy denture at 100 *μ*m/layer ((d) occlusal surface of upper denture, (e) mucosal surface of upper denture, (f) occlusal surface of lower denture, and (g) mucosal surface of lower denture).

**Figure 3:**
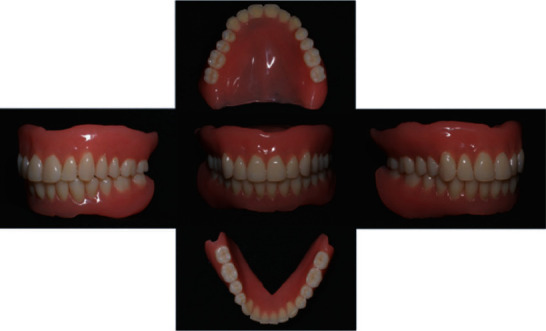
New dentures.

**Figure 4:**
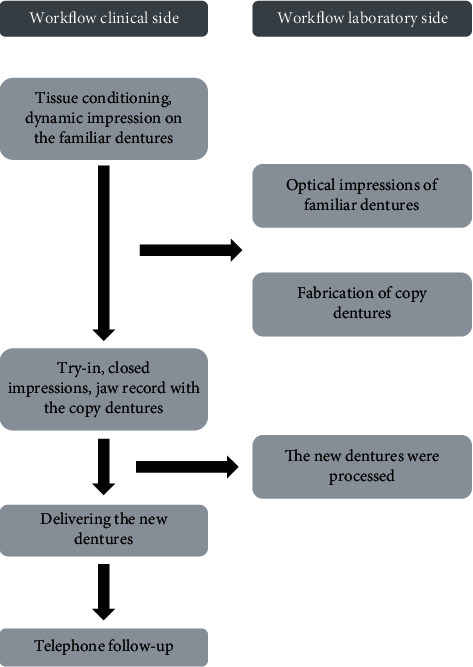
Flowchart of the combined workflow.

1. First clinical stage: the minimum amount of tissue conditioner required (Tissue Conditioner II, Shofu, Inc., Kyoto, Japan) was used to correct the poor fit of the old dentures and to record dynamic impressions of the dentures, following which minor occlusal alterations were undertaken. One week later, a follow-up teleconsultation of the patient revealed disappearance of pain in the mucosa of the jaw ridge during meals and an increase in the food intake. We asked the patient's family (her son) to bring her upper and lower dentures to our hospital to record optical impressions for the upper and lower dentures using an optical scanner (Neway; Open Tech 3D s.r.l., Rezzato, Brescia, Italy) (Figures 2(a) and 2(b)); the dentures were returned to the patient immediately
2. First laboratory stage: the scanned data were converted into standard tessellation language (STL) data and exported to a three-dimensional (3D) printer interface software (Composer; Asiga, Sydney, Australia) (Figure 2(c)), and then a 3D printer (Asiga MAX UV; Asiga) using dental photocurable resin (Freeprint Tray UV; Detax, Ettlingen, Germany) which fabricated the copy denture at 100 *μ*m/layer. After washing the copy dentures with isopropyl alcohol, final polymerization was performed using a dental polymerization apparatus (Asiga Flash; Asiga). The copy dentures were polished with a dental carbide cutting tool (H251GSQ-060; Komet, Lemgo, Germany) (Figures 2(d)–2(g)). 3D scanning of each of the upper and lower dentures according to the software's instructions and saving of the STL data were completed in 10 min. An additional 1 min was required for transfer of the STL data to a composer. The time required for setting the support bars in the composer was 5 min; printing, 75 min; washing, 6 mins; final polymerization, 30 min; and polishing, 10 min. The total laboratory work required approximately 2.5 h
3. Second clinical stage: the copy dentures were slightly adjusted to ensure the correct occlusal contact. A closed mouth impression was recorded using the minimum required amount of a light-body wash impression (Fusion II Extra Wash Type, GC Corp. Tokyo. Japan) in the copy dentures for both the maxillary and mandibular bases inside the patient's mouth. While taking the impressions, the patient was asked to sit upright and close her mouth. The jaw record was obtained at the same time
4. Second laboratory stage: the impressions were cast and articulated, the morphology of the polishing surface of the copy dentures were converted into wax rims utilizing plaster masks, and teeth were set on those rims. After deflasking, the dentures were checked using the articulator for processing errors and subsequently polished (Figure 3).
5. Third clinical stage: we visited the patient's home and delivered the completed dentures following an intraoral check. The fit and occlusion of the dentures were good
6. Fourth clinical stage: telephonic follow-up was performed twice every other week, and there was no complaint of pain during meals; an increase in food intake and a decrease in the duration of meals were noted. The procedures followed in this case are shown in Figure 4. Only two visits were required for taking the impression and delivering the complete dentures. In addition, the patient's dentures were sent to the dental office after her dinner and returned to her the next morning, thus avoiding disturbance of the patient's normal eating routine

## 4. Discussion

Recording oral impressions in elderly individuals requiring long-term home care is often challenging. Although dentists have tried various measures, such as the use of impression materials with low fluidity and a short curing time, the risk of accidental ingestion and aspiration during impression taking has been reported in elderly patients [2]. On the other hand, intraoral scanners have significantly improved with the aim of providing highly safe and effective dental treatment over the last decade. For example, a previous study examined the patient's comfort and stress levels while taking oral impressions digitally and conventionally in patients with gag reflex or breathing difficulty; the study revealed that the intraoral scanner scored better than the conventional method [6]. Another previous study found no significant difference in the accuracy between dental prostheses manufactured using an alginate impression and those made using an intraoral scanner [5]. However, certain challenges remain with the use of intraoral scanners. It is impossible to record an impression of areas that are outside the scanning range, such as undercuts of ridges and deep grooves. The accuracy of the impression is also affected by moisture, including salivary contamination [7–11]. Moreover, a digital jaw relation record can be obtained in edentulous patients [12]. However, the success or failure of the scan is seemingly dependent on the operator's technique. Therefore, at present, it is practical to incorporate digital dental technology into the manufacturing process of the conventional method for improvement of safety and efficiency of the dental treatment, rather than using a digital workflow for all the processes. In the present case, for a patient with dementia with difficulty communicating, incorporating digital dental technology into the manufacturing process of the conventional method enabled us to provide high-quality complete dentures safely because of the following reasons.

First, acceptance of new dentures in this case appeared relatively simple owing to the use of dentures that copied the morphology of a familiar denture that the patient had been using for a long time. In contrast, previous studies reported that older patients have difficulty accepting new dentures [13]. Dentists have been trying to determine a technique for transferring the morphology of familiar dentures to new dentures. For example, the neutral zone technique [14] uses impressions of the polishing surface of the denture followed by boxing using a silicone putty material to obtain the tongue and facial matrices. Similar to the method used in this case, the polished surface morphology can be transferred easily using optical scanning. Moreover, we could obtain 3D data of the familiar dentures in case the patient loses or breaks the dentures in the future; in such cases, the 3D data of the dentures could be used for new denture fabrication.

Second, only two visits were required for taking the impression and delivering the complete dentures, reducing the quantity of impression material used and frequency of impressions taken. Many such patients who require long-term care, especially those with moderate or severe dementia, refuse long-duration or highly invasive treatment [15]; therefore, selection of a simple and minimally invasive treatment for such patients is desirable.

Finally, the reduction in contact and time spent between patients and healthcare professionals owing to the use of digital dental technology could help reduce the risk of spread of the new coronavirus disease (COVID-19), which is currently present worldwide. According to public health authorities, significant exposure is defined as a “face to face contact within 2 m with a patient with symptomatic COVID-19” in the range of a few minutes up to 30 min [16]. Therefore, conventional “face to face” dental treatment, which increases the risk of acquiring infection, should be minimized.

## 5. Conclusion

Our case of a patient with dementia with difficulty in communication, incorporating digital dental technology into the manufacturing process of the conventional method, enabled us to provide high-quality complete denture safely, due to the following reasons. First, acceptance of new dentures was enhanced by using dentures that copied the morphology of a familiar denture. Second, only two visits were required for recording the impression and delivering the complete dentures. Moreover, the patient's dentures were brought to the dental office by her son after her dinner and returned to her son immediately, thus avoiding any disturbance to her eating routines. These factors reduced the burden on the patient and her family.

## References

[1] Qiu W. Q., Dean M., Liu T. (2010). Physical and mental health of homebound older adults: an overlooked population. *Journal of the American Geriatrics Society*.

[2] Morita H., Hashimoto A., Inoue R., Yoshimoto S., Yoneda M., Hirofuji T. (2016). Successful Fitting of a Complete Maxillary Denture in a Patient with Severe Alzheimer’s Disease Complicated by Oral Dyskinesia. *Case Reports in Dentistry*.

[3] Nicolas E., Lassauzay C., Pickering G., Croze J., Hennequin M. (2008). Needs in screening cardiovascular parameters during dental care in the elderly. *Aging Clinical and Experimental Research*.

[4] Suese K. (2020). Progress in digital dentistry: the practical use of intraoral scanners. *Dental Materials Journal*.

[5] Tsirogiannis P., Reissmann D. R., Heydecke G. (2016). Evaluation of the marginal fit of single-unit, complete-coverage ceramic restorations fabricated after digital and conventional impressions: a systematic review and meta-analysis. *The Journal of Prosthetic Dentistry*.

[6] Mangano A., Beretta M., Luongo G., Mangano C., Mangano F. (2018). Conventional vs digital impressions: acceptability, treatment comfort and stress among young orthodontic patients. *The Open Dentistry Journal*.

[7] Kurz M., Attin T., Mehl A. (2015). Influence of material surface on the scanning error of a powder-free 3D measuring system. *Clinical Oral Investigations*.

[8] Arakida T., Kanazawa M., Iwaki M., Suzuki T., Minakuchi S. (2018). Evaluating the influence of ambient light on scanning trueness, precision, and time of intra oral scanner. *Journal of Prosthodontic Research*.

[9] Revilla-León M., Jiang P., Sadeghpour M. (2020). Intraoral digital scans--Part 1: Influence of ambient scanning light conditions on the accuracy (trueness and precision) of different intraoral scanners. *The Journal of Prosthetic Dentistry*.

[10] Revilla-León M., Jiang P., Sadeghpour M. (2020). Intraoral digital scans: Part 2--influence of ambient scanning light conditions on the mesh quality of different intraoral scanners. *The Journal of Prosthetic Dentistry*.

[11] Revilla-León M., Subramanian S. G., Özcan M., Krishnamurthy V. R. (2020). Clinical study of the influence of ambient light scanning conditions on the accuracy (trueness and precision) of an intraoral scanner. *Journal of Prosthodontics*.

[12] Kanazawa M., Iwaki M., Arakida T., Minakuchi S. (2018). Digital impression and jaw relation record for the fabrication of CAD/CAM custom tray. *Journal of Prosthodontic Research*.

[13] Bergman B., Carlsson G. E. (1972). Review of 54 complete denture wearers. Patients’ opinions 1 year after treatment. *Acta Odontologica Scandinavica*.

[14] Ikebe K., Okuno I., Nokubi T. (2006). Effect of adding impression material to mandibular denture space in piezography. *Journal of Oral Rehabilitation*.

[15] Fiske J., Frenkel H., Griffiths J., Jones V., British Society of Gerodontology, British Society for Disability and Oral Health (2006). Guidelines for the development of local standards of oral health care for people with dementia. *Gerodontology*.

[16] Klompas M., Morris C. A., Sinclair J., Pearson M., Shenoy E. S. (2020). Universal masking in hospitals in the COVID-19 era. *The New England Journal of Medicine*.

